# Disparities in inflammation between non-Hispanic black and white individuals with lung cancer in the Greater Chicago Metropolitan area

**DOI:** 10.3389/fimmu.2022.1008674

**Published:** 2022-12-05

**Authors:** Cecily A. Byrne, Sandra L. Gomez, Sage Kim, Vanessa M. Oddo, Timothy J. Koh, Giamila Fantuzzi

**Affiliations:** ^1^ Department of Kinesiology and Nutrition, University of Illinois Chicago, Chicago, IL, United States; ^2^ Department of Clinical Nutrition, Rush University Medical Center, Chicago, IL, United States; ^3^ School of Public Health, University of Illinois Chicago, Chicago, IL, United States

**Keywords:** inflammation, racial disparities, neighborhood disadvantage, neutrophil/lymphocyte ratio, lung cancer

## Abstract

**Background:**

Lung cancer incidence and mortality rates are higher in Non-Hispanic Black (NHB) compared to Non-Hispanic White (NHW) individuals in the Chicago metropolitan area, which may be related to exposure to chronic stress which may increase inflammation.

**Specific aim:**

We investigated disparities in inflammation as measured by neutrophil to lymphocyte ratio (NLR) in individuals with lung cancer by race and by neighborhood concentrated disadvantage index (CDI).

**Methods:**

This retrospective, cross-sectional study included 263 NHB and NHW adults with lung cancer. We analyzed NLR as a continuous and categorical variable to determine degree and prevalence of inflammation. We used Mann Whitney U, t-tests, Chi square tests, linear and logistic regression models as appropriate.

**Results:**

More than 60% of subjects had inflammation (NLR ≥ 3) at lung cancer diagnosis. The degree of inflammation was significantly lower in NHB (NLR 5.50 +/- 7.45) compared to NHW individuals (NLR 6.53 +/- 6.53; p=0.01) but did not differ by neighborhood CDI. The prevalence of inflammation (NLR ≥ 3) was significantly lower in NHB (55.07%) compared to NHW individuals (71.20%; p<0.01) and in those from the most disadvantaged (54.07%) compared to the least disadvantaged (71.88%; p<0.01) neighborhoods.

**Conclusion:**

At lung cancer diagnosis, there is a lower degree and prevalence of inflammation in NHB compared to NHW individuals, and lower prevalence in those residing in the most disadvantaged neighborhoods. Further research is needed to determine mechanisms of inflammation that may be contributing to lung cancer disparities as well as whether NLR is an appropriate biomarker when examining racial differences in inflammation.

## Introduction

Lung cancer is one of the deadliest cancers, with similar incidence and mortality rates nationwide for Non-Hispanic Black (NHB) and Non-Hispanic White (NHW) individuals (1). However, in Illinois, and specifically in Cook County, there is a stark disparity in lung cancer incidence and mortality rates between NHB and NHW individuals. In 2014-2018, the incidence for NHB was 71.9/100,000 compared to 54.3/100,000 for NHW individuals, and the mortality rate was 50.1/100,000 for NHB versus 34.4/100,000 for NHW individuals (1). The reasons for these racial disparities may be related to chronic social stress resulting from racism, perceived discrimination, and socioeconomic disadvantage, as well as segregation in Cook County; each of these factors can increase systemic inflammation, a critical contributor to cancer pathogenesis (2, 3). However, studies have yet to investigate disparities in inflammation in subjects with lung cancer by race and by socioeconomic factors.

Chronic systemic inflammation associated with smoking (4) and other conditions such as chronic obstructive pulmonary disease (5) plays a critical role in the development of lung cancer (6). Inflammation is an independent predictor of mortality in lung cancer, associated with decreased progression-free and overall survival (7, 8). Systemic inflammation can be measured *via* circulating levels of various biomarkers, such as C-reactive protein (CRP), and cytokines like interleukin-6 (IL-6) (9, 10). However, these biomarkers are not routinely measured in the clinical setting (11). Alternatively, neutrophil to lymphocyte ratio (NLR) is a marker of systemic inflammation derived from absolute neutrophil and lymphocyte counts performed during complete blood counts, parameters that are routinely available in medical records. During the systemic inflammatory response, there are alterations in the numbers of circulating white blood cells, including neutrophilia and lymphocytopenia, resulting in an increase in NLR. An elevated NLR is a prognostic indicator of decreased survival in individuals with solid tumors, including lung cancer (9, 12–14).

Race is a social rather than a biological construct used to oppress and dominate one group over another, resulting in racism and discrimination (15). Socioeconomic status (SES) is a structural determinant of health inequities (16) and a fundamental cause of disease (17). Racial inequalities in health reflect, in part, differences in SES, including income, education, occupation, wealth, working and housing conditions (15). Moreover, an individual’s neighborhood environment also impacts health, independent of individual SES (18, 19). Concentrated disadvantage, often quantified through the concentrated disadvantage index (CDI), describes the degree of neighborhood economic and social disadvantage (20, 21), especially the geographic isolation of poor, Black, and single-parent families and the bundling of social issues, including crime, physical and social disorder, and school dropout (22). Individuals from disadvantaged neighborhoods are exposed to physical (e.g., vacancies, pollution, disinvestment) and social (e.g., violence, segregation) stressors which can impact health through stress (23–25) and lead to increased inflammation (2, 26–28).

While several studies evaluated inflammation in NHB versus NHW individuals both in the general population and in individuals with cancer, the results are inconsistent, showing either higher or lower inflammation in NHB versus NHW individuals (2, 29–37). On the other hand, only a few studies have investigated the degree of inflammation by neighborhood concentrated disadvantage or neighborhood SES. Those studies show higher inflammation, as measured by CRP, in the most disadvantaged or low SES neighborhoods (38–41).

Because of inconsistencies in the literature on inflammatory biomarkers by race and because of lack of data specifically in lung cancer, we aimed to investigate disparities in inflammation as measured by NLR in subjects with lung cancer by race. A secondary aim was to investigate disparities in inflammation as measured by NLR in subjects with lung cancer by neighborhood CDI. We hypothesized that NHB and individuals from disadvantaged neighborhoods would have higher NLR due to chronic stress.

## Materials and methods

### Study population and setting

This retrospective, cross-sectional study utilized a convenience sample from a parent study of 1251 lung cancer cases from an urban, tertiary medical center in Cook County, IL that were diagnosed or treated between 2014 and 2016. The number of subjects for data extraction was based on pilot data and *a priori* power calculations (90% power, alpha=0.05) to test for differences in NLR by race (n=72, 36 NHB and 36 NHW), as well as obtaining an equal representation of NHB and NHW subjects by sex. Approximately half (n=604) of the lung cancer cases were reviewed, and subjects were included in this study based on the following criteria: being aged 19 years or older, diagnosed with lung cancer, having a diagnostic CT scan available, and self-identifying as NHB or NHW (n=293). Three hundred eleven subjects were excluded due to other race (n=117), no lung cancer diagnosis (n=5), no diagnostic CT scan available (n=180), and duplicate or restricted records (n=9). Subjects were additionally excluded if they were missing data for NLR, the primary dependent variable (n=14), cancer stage was unavailable (n=1), or their residential addresses were outside IL (n=15) for a final sample size of n=263. See **Supplementary Figure 1** for details on how the sample was derived.

### Data extraction

Electronic medical records (EMR) were retrospectively reviewed, and demographic and clinical information was collected relative to cancer diagnosis date. Clinical and demographic data extracted included age, height, weight, race, ethnicity, sex, tumor type, clinical stage of cancer, date of cancer diagnosis, neutrophil count, lymphocyte count, and residential address at cancer diagnosis. Race, ethnicity, and sex were self-identified. Body mass index (BMI) was calculated as weight in kilograms/height in meters squared; BMI was categorized into underweight (BMI <18.5 kg/m^2^), normal (BMI >/= 18.5 and <25 kg/m^2^), overweight (BMI >/= 25 and <30 kg/m^2^) and obese (BMI > /=30 kg/m^2^) status. Stage of cancer was categorized as early-stage (in situ, stage 1 and stage 2) versus late-stage (stage 3 and 4). Lab values collected were within 30 days of cancer diagnosis, closest to the diagnosis date, and before any treatment (chemotherapy, radiation, or surgery) for lung cancer was initiated.

### Primary dependent variable

The primary study outcome was inflammation as defined by NLR, which was computed as the ratio of total neutrophil to total lymphocyte count. We investigated both the degree of inflammation in individuals using NLR as a continuous variable and the prevalence of inflammation in the population as defined by NLR ≥3 (moderate to high inflammation), as an elevated NLR is pathological and associated with decreased overall survival, cancer-specific survival, and progression-free survival in patients with solid tumors (9, 13, 42, 43).

### Primary independent variables

Race (NHB and NHW) served as our primary independent variable and was self-reported. Secondarily, we investigated disparities in inflammation in subjects with lung cancer by neighborhood CDI as an exploratory analysis. To calculate CDI, residential addresses were geocoded using ArcGIS Desktop 10.8.1 (ESRI https://www.esri.com/en-us/home). We then retrieved census tract level neighborhood characteristics from the U.S. Census American Community Survey (ACS) 5-year estimates from 2013-2017 as this survey spanned the years for subjects’ cancer diagnosis. Principal component analysis was conducted to compute neighborhood CDI using: % individuals living below poverty, % individuals with less than high school education, % female headed households with dependent children, % unemployed among working-age individuals, and median household income (20). Higher CDI scores represent greater disadvantage, and because CDI is a standardized composite score, the mean was zero and the standard deviation was 1 (20, 44). In this sample, the CDI ranged from -1.82 to 3.73, and a binary variable for CDI (least versus most disadvantaged neighborhoods) was created with least disadvantaged defined as </= 50%ile (including median of 0.161468) and most disadvantaged defined as >50%ile for CDI. The neighborhoods were also categorized by racial composition with majority Black neighborhoods defined as ≥75% Black and majority White neighborhoods defined as ≥ 75% White (20).

### Statistical analysis

Standard descriptive statistics (N [%] or mean [standard deviation] were calculated and statistical differences were estimated using Mann Whitney U (for NLR, which is not normally distributed) or t-test for continuous variables and Pearson’s chi-square test for categorical variables. NLR was log-transformed to reduce skewness and normalize the distribution. Prevalence of inflammation by race and neighborhood CDI was analyzed by setting the threshold for inflammation as NLR ≥3 (9, 13, 42, 43) according to published data. On the other hand, NLR<3 was categorized as no inflammation (9, 13, 42, 43, 45, 46). We used linear (degree) and logistic (prevalence) regression to test whether clinical and demographic characteristics were associated with NLR in univariate and multivariable analysis. Forward, backward, and stepwise modeling procedures were completed to determine significant predictors (race, neighborhood CDI, stage of cancer, BMI (categorical variable), age (continuous and categorical variable), smoking and alcohol history, racial composition of the neighborhood) to include in the linear and logistic regression models. Interactions between race and CDI and between race and stage of cancer were tested for significance. Statistical significance was defined as p<0.05. All statistical analyses were conducted using SAS^®^ Studio, SAS^®^ OnDemand for Academics (Copyright^©^ 2021, SAS Institute Inc., Cary, NC, USA). This study was approved by the University of Illinois Chicago (Protocol #2020-0677) and the Rush University Medical Center (ORA #18013002-IRB01) Institutional Review Boards.

## Results

### Overall demographics of subjects

The sample consisted of a similar number of males (n=137; 52.1%) and females (n=126; 47.9%) with a mean age of 69.2 +/- 9.9 years and a BMI of 26.3 +/- 5.7 kg/m^2^ (see **Supplementary Table 1** for details about BMI distribution). By design, half of the subjects were NHB (52.5%) and half were NHW (47.5%). A significantly higher number of subjects had late-stage cancer (58.9%) compared to early-stage cancer (41.1%; p<0.004). A significantly higher number of subjects had non-small cell lung cancer (NSCLC) versus small cell lung cancer (SCLC) or other sub-types/unknown (85.2% versus 2.7% and 12.2%, respectively; p<0.0001).

### Demographics by race

As shown in **Table 1**, age and BMI did not differ significantly between NHB and NHW individuals. A significantly higher proportion of NHB (71.0%) had late-stage lung cancer compared to NHW individuals (45.6%) but the histology of lung cancer did not differ by race. As expected in a population of subjects with lung cancer, the vast majority of individuals (92.0%) were current or former smokers, with comparable rates in NHB and NHW individuals. Significantly more NHB (75.4%) lived in the most disadvantaged neighborhoods compared to NHW individuals (24.8%). Moreover, 66% of NHB individuals lived in neighborhoods with ≥75% Blacks compared to 34% living in neighborhoods with <75% Blacks (**Supplementary Table 1**). Refer to **Supplementary Table 2** for demographics by neighborhood CDI.

**Table 1:** Demographics, inflammatory biomarkers, and neighborhood socioeconomic variables of subjects with lung cancer overall and by race (n=263)^1^.

	Overall Sample		NHB(n=138)	NHW(n=125)	p-value
Age (years)	69.17 +/- 9.92		68.54 +/- 10.47	69.86 +/- 9.04	0.28
BMI (kg/m^2^)	26.26 +/- 5.74		25.91 +/- 6.17	26.66 +/- 5.21	0.29
NLR	5.99 +/- 7.03		5.50 +/- 7.45	6.53 +/- 6.53	0.01*
Sex					
Male	137 (52.09)	0.50	72 (52.17)	65 (52.00)	0.98
Female	126 (47.91)		66 (47.83)	60 (48.00)	
Cancer Stage					
Early-Stage (0,1,2)	108 (41.06)	0.004	40 (28.99)	68 (54.40)	<0.0001
Late-Stage (3, 4)	155 (58.94)		98 (71.01)	57 (45.60)	
Histology		<0.0001			0.35
NSCLC	224 (85.17)		120 (86.96)	104 (83.20)	
SCLC	7 (2.66)		5 (3.62)	2 (1.60)	
Unknown/Other	32 (12.17)		13 (9.42)	19 (15.20)	
Smoking History					
Current/Former	240 (91.95)	<0.0001	126 (92.65)	114 (91.20)	0.67
Never	21 (8.05)		10 (7.35)	11 (8.80)	
CDI					
Least Disadvantaged Neighborhoods	128 (48.67)	0.67	34 (24.64)	94 (75.20)	<0.0001
Most Disadvantaged Neighborhoods	135 (51.33)		104 (75.36)	31 (24.80)	

^1^Values are means +/- SDs or n (%). *Mann Whitney U test as NLR is not normally distributed in this sample. T-test used for other continuous variables and Pearson’s chi square test used for categorical variables. NHB,Non-Hispanic Black; NHW, Non-Hispanic White; BMI, body mass index; kg, kilograms; m^2^, meters squared; NLR, neutrophil to lymphocyte ratio; CDI, concentrated disadvantage index; NSCLC, non-small cell lung cancer; SCLC, small cell lung cancer.

### Degree of inflammation

Overall, subjects included in this study experienced a substantial degree of inflammation at diagnosis based on NLR (5.99 +/- 7.03) (see **Table 1**). Before controlling for any potential confounders, we found a significantly lower NLR in NHB compared to NHW (5.50 +/- 7.45 vs 6.53 +/- 6.53; p=0.01) individuals. Moreover, there was no significant difference in NLR when data was analyzed by neighborhood CDI, though NLR trended higher in those from the most disadvantaged neighborhoods compared to those from the least disadvantaged ones. Analyzing log-transformed NLR by race and CDI did not change the results. NLR was also analyzed by CDI quartiles, and again there was no signficant difference when comparing NLR in the least disadvantaged quartile versus the most disadvantaged quartile (see **Supplementary Table 2**). The data was then stratified by race and NLR was analyzed by neighborhood CDI to detect differences in NHB and in NHW individuals separately. There was no significant difference in NLR by neighborhood CDI in either NHB or NHW individuals after stratifying by race (See **Supplementary Table 3**).

In addition and as expected (14, 35, 43, 47), NLR was significantly higher in late-stage versus early-stage lung cancer (6.67 +/- 8.02 versus 5.01 +/- 5.17; p=0.04). When stratifying by race, NLR was significantly lower in NHB compared to NHW individuals in both early and late stages of lung cancer (early-stage: 3.58 +/- 2.59 vs 5.86 +/- 6.07 in NHB vs NHW, respectively; p=0.02; late-stage: 6.29 +/- 8.57 vs 7.34 +/- 7.00 in NHB vs NHW, respectively; p=0.03). We also analyzed NLR by histology sub-type of lung cancer. Again, there was no signficant difference in NLR when looking at NSCLC versus SCLC (5.72 +/- 6.53 versus 8.85 +/- 10.58, respectively; p=0.24) nor when looking at NSCLC versus all other sub-types (5.72 +/- 6.53 versus 7.52 +/- 9.46, respectively; p=0.15. When looking only at those with a current or former history of smoking (n=242), NLR was still significantly lower in NHB compared to NHW individuals (5.06 +/- 5.99 versus 6.66 +/- 6.73; p=0.006) and in those from the most disadvantaged compared to the least disadvantaged neighborhoods (5.61 +/- 7.05 versus 6.03 +/- 5.63; p=0.04). See **Supplementary Table 4** for additional NLR analyses.

**Table 2** details the association between demographics, clinical characteristics and NLR. In multivariable analysis, NHB race was significantly associated with a lower NLR (-1.95 units) compared to NHW after controlling for stage of cancer, BMI category, age category and smoking history (p=0.01). In the fully adjusted model, NHB race was significantly associated with a 2.22 unit decrease in NLR compared to NHW after controlling for neighborhood CDI, stage of cancer, BMI category, age category, and smoking history. Using the log-transformed NLR in multivariate linear regression analysis made no difference in the association between NLR and race or neighborhood CDI.

**Table 2:** Univariate and adjusted linear regression estimates of the association between demographic and clinical characteristics and NLR.

		Univariate		Multivariate – Model 1*		Multivariate – Model 2**	
Variable	Category	Coefficient (95% CI)	p-value	Coefficient (95% CI)	p-value	Coefficient (95% CI)	p-value
Race	NHW	Ref					
	NHB	-1.03 (-2.74, 0.67)	0.23	-1.95 (-3.51, -0.40)	0.01	-2.22 (-4.00, -0.44)	0.01
CDI (Categorical)	Least Disadvantaged	Ref					
	Most Disadvantaged	0.08 (-1.63, 1.79)	0.93			0.56 (-1.23, 2.35)	0.54
Stage of Cancer	Early-Stage	Ref					
	Late-Stage	1.66 (-0.07, 3.39)	0.06	1.61 (-0.01, 3.24)	0.05	1.55 (-0.08, 3.19)	0.06
Smoking history							
	Never	Ref					
Age	Current/former	0.16 (-2.65, 2.97)	0.91	0.10 (-2.71, 2.91)	0.94	0.08 (-2.73, 2.90)	0.95
	<65 years	Ref					
	>65 years	-0.90 (-2.72, 0.91)	0.33	-0.09 (-1.76, 1.57)	0.91	-0.03 (-1.71, 1.65)	0.97
BMI	underweight	5.09 (2.07, 8.12)	0.001	2.47 (-0.55, 5.49)	0.11	2.54 (-0.49, 5.58)	0.10
	Normal	Ref					
	overweight	-1.35 (-3.13, 0.42)	0.13	-1.02 (-2.81, 0.78)	0.27	-1.03 (-2.83, 0.77)	0.36
	obese	-0.91 (-2.99, 1.16)	0.39	-0.90 (-3.00, 1.30)	0.40	-0.98 (-3.09, 1.14)	0.36

NHB, Non-Hispanic Black; NHW, Non-Hispanic White; NLR, neutrophil to lymphocyte ratio; CDI, concentrated disadvantage index; BMI, body mass index *Model 1- controlling for stage of cancer, age category, BMI category, and smoking history **Model 2- controlling for neighborhood CDI, stage of cancer, age category, BMI category, and smoking history.

Collectively, these data indicate a lower degree of inflammation in NHB compared to NHW individuals when using NLR as a biomarker.

### Prevalence of inflammation by race and by neighborhood CDI

Approximately two thirds of subjects with lung cancer (62.7%) presented with inflammation at diagnosis when using a threshold value of NLR ≥ 3. Prevalence of inflammation was significantly lower in NHB compared to NHW individuals (55.1% vs 71.2%; p=0.007).

There was also a significantly lower prevalence of individuals with NLR ≥ 3 from the most disadvantaged neighborhoods compared to those from the least disadvantaged neighborhoods (54.1% vs 71.9%; p=0.003). Collectively, these data indicate a lower prevalence of inflammation in NHB compared to NHW individuals and in the most disadvantaged compared with the least disadvantaged neighborhoods when using NLR as a biomarker and before adjusting for potential confounders.

The data was then stratified by race to determine if inflammation prevalence differed by neighborhood CDI in NHB and in NHW individuals separately. In NHB individuals, there were no significant differences in inflammation prevalence by neighborhood CDI. In NHW individuals, inflammation prevalence for NLR ≥ 3 was lower in the most disadvantaged compared to the least disadvantaged neighborhoods but this did not reach statistical significance (58.06% vs 75.53%; p=0.062).

**Table 3** details the association between demographics and clinical characteristics and inflammation prevalence (NLR ≥ 3). In the fully adjusted model, NHB race was associated with 51% lower odds (95% Confidence Interval [CI]: 0.26, 0.91) of inflammation prevalence after controlling for neighborhood CDI, stage of cancer, age and BMI categories, and smoking history (p=0.02). Those living in the most disadvantaged neighborhoods had 43% lower odds (95% CI: 0.31, 1.06) of inflammation compared to those living in the least disadvantaged neighborhoods after controlling for race, stage of cancer, age and BMI categories, and smoking history but this did not reach statistical significance (p=0.08). As expected, late-stage lung cancer was associated with 1.99 times higher odds of inflammation (95% CI: 1.11, 3.59) when compared to early-stage lung cancer after controlling for race, neighborhood CDI, age and BMI categories, and smoking history. Finally, underweight status was associated with 5.2 times higher odds of inflammation (95% CI: 1.10, 24.66) compared to normal weight status (p=0.04) in the fully adjusted model.

**Table 3:** Univariate and adjusted logistic regression estimates of the association between demographic and clinical characteristics and inflammation prevalence (NLR ≥ 3).

Variable	Category	Univariate Analysis		Multivariate Analysis- Model 1*		Multivariate Analysis- Model 2**	
		OR (95% CI)	p-value	OR (95% CI)	p-value	OR (95% CI)	p-value
Race	NHW	Reference					
	NHB	0.50 (0.30, 0.83)	0.007	0.38 (0.22, 0.67)	0.0008	0.49 (0.26, 0.91)	0.02
CDI (Categorical)	Least Disadvantaged	Reference					
	Most Disadvantaged	0.46 (0.28, 0.77)	0.003			0.57 (0.31, 1.06)	0.08
Stage of Cancer	Early-Stage	Reference					
	Late-Stage	1.47 (0.89, 2.44)	0.14	1.84 (1.04, 3.28)	0.04	1.99 (1.11, 3.59)	0.02
Smoking history							
	Never	Ref					
	Current/former	1.04 (0.42, 2.62)	0.93	0.99 (0.37, 2.64)	0.98	0.99 (0.36, 2.69)	0.98
Age	<65 years	Reference					
	>65 years	1.12 (0.66, 1.91)	0.67	1.23 (0.68, 2.20)	0.49	1.15 (0.64, 2.08)	0.64
BMI	underweight	3.38 (0.93, 12.33)	0.06	5.46 (1.16, 25.71)	0.03	5.20 (1.10, 24.66)	0.04
	normal	reference					
	overweight	0.88 (0.48, 1.60)	0.66	0.91 (0.49, 1.68)	0.75	0.91 (0.49, 1.70)	0.77
	obese	0.55 (0.28, 1.09)	0.09	0.54 (0.27, 1.11)	0.09	0.58 (0.28, 1.20)	0.14

NHB, Non-Hispanic Black; NHW, Non-Hispanic White; NLR, neutrophil to lymphocyte ratio; CDI, concentrated disadvantage index; BMI, body mass index *Model 1- controlling for stage of cancer, age category, BMI category, and smoking history **Model 2- controlling for neighborhood CDI, stage of cancer, age category, BMI category, and smoking history.

## Discussion

Our study found that the degree and prevalence of inflammation, as measured by NLR as a continuous variable and NLR ≥ 3, respectively, was significantly lower in NHB compared to NHW individuals with lung cancer. Inflammation prevalence (NLR ≥ 3) was also significantly lower in patients with lung cancer who resided in the most disadvantaged neighborhoods compared to those from the least disadvantaged neighborhoods.

In agreement with previous reports (7, 8), we found that inflammation is both present and prevalent in lung cancer, with over 60% of subjects presenting with inflammation at diagnosis and higher NLR values for both NHB and NHW individuals with lung cancer compared to previously reported baseline NLR values for the general population obtained from NHANES data, which excluded subjects with cancer (34). We also confirm that inflammation was significantly more severe in late-stage compared to early-stage lung cancer (14, 35, 43, 47). In our study, NLR was significantly higher in those who were underweight at cancer diagnosis (with significantly more late-stage versus early-stage lung cancer) compared to those who were normal weight, overweight or obese, consistent with a study that found significantly higher NLR in underweight individuals after treatment for ovarian cancer compared to obese individuals (48).

We found a significant difference in the degree of inflammation as measured by NLR by race, with lower values in NHB compared to NHW individuals at lung cancer diagnosis. Our results are consistent with a study that included patients with various types of cancer but not lung cancer, and a study in locally advanced non-small cell lung cancer (NSCLC), both of which found that pre-treatment NLR was significantly lower in NHB versus NHW individuals (35, 49). The significantly lower NLR in NHB individuals is also consistent with two NHANES studies that found significantly lower NLR in NHB compared to NHW individuals in the general population (34, 36). Benign Ethnic Neutropenia (BEN), a condition present in individuals of African, Caribbean, Middle Eastern, and West Indian descent, may help explain this discrepancy. In BEN, low neutrophil counts (less than 1500/microliter) are present in the absence of any identifiable causes or consequences (50). If NHB individuals have BEN or a lower NLR value at baseline, then NLR may not reach the high levels seen in NHW individuals at lung cancer diagnosis despite an increase from baseline levels (34).

Our results, showing no differences in the degree of inflammation by neighborhood CDI, are in contrast with several studies that investigated the degree of inflammation (measured by CRP) by neighborhood CDI or neighborhood SES, finding that CRP decreased with increasing neighborhood SES and a positive correlation between CDI and CRP (38–41). Our contrasting findings may be related to the presence or absence of lung cancer, though the use of different biomarkers -NLR vs CRP - is also likely a critical parameter. Indeed, NLR may only be weakly correlated with CRP, and there may be different mechanisms driving changes in these biomarkers (36). Importantly, CRP is not routinely measured in healthcare settings (11), whereas NLR can be calculated from a routine complete blood count, thus allowing its use in retrospective studies that utilize EMR.

When looking at the prevalence of inflammation in the population as quantified by NLR ≥ 3, we found significantly lower values in NHB compared to NHW individuals and in the most disadvantaged neighborhoods compared to the least disadvantaged ones. These data are in agreement with a study evaluating the prevalence of inflammation in hepatocellular carcinoma (indicated by NLR>4) (47) and in colorectal cancer (NLR ≥3) (43), which found that NHB individuals had a lower prevalence of elevated NLR compared to NHW, and that individuals with a lower SES were also less likely to have elevated NLR compared to those with a higher SES (47). In contrast, studies evaluating prevalence of inflammation, as indicated by CRP, by neighborhood deprivation or “risky” neighborhoods found the highest prevalence of inflammation in adults and children in the most deprived or “riskiest” neighborhoods (26–28). While these studies contrast with our findings, the discrepant results may be related to heterogeneity in the definition of neighborhood deprivation or SES, to the inclusion of subjects with lung cancer in our study versus the general population in previous reports, to issues such as adiposity or levels of physical activity, but also to the biomarker measured (NLR vs CRP). Indeed, the lower prevalence of inflammation among NHB individuals that we found may be due in part to having baseline NLR lower than NHW individuals in the absence of cancer and the need for a lower NLR threshold to define the presence of inflammation in NHB individuals (34). If NHB individuals have a mean NLR that is significantly lower than NHW individuals in the absence of cancer, this may indicate that cut-off values for increased NLR in chronic disease, including cancer, may differ by race and should therefore be adjusted. NLR >3 is pathological and reflects innate versus adaptive immunity (13); however, it is possible that this NLR level cannot be reached by NHB individuals or reflects more severe inflammation in NHB versus NHW individuals (34).

It’s also important to note that more NHB and those from disadvantaged neighborhoods presented with late-stage lung cancer, and that stage of cancer impacted the degree of inflammation, with significantly higher NLR in late-stage versus early-stage lung cancer. It would, therefore, be expected that NHB individuals would present with higher NLR at diagnosis, as they presented with more advanced cancer. However, our study did not confirm this expectation. In fact, after controlling for stage of cancer, neighborhood CDI, BMI and age categories, and smoking history, NHB individuals had 51% lower odds of inflammation prevalence and lower mean NLR compared to NHW individuals, which may instead suggest a lower physiological stress response and immunosuppression, as elevated NLR is an indicator of neuroendocrine stress (13). During times of stress and increased cortisol production, there is a shift from adaptive to innate immunity with lymphocytopenia and neutrophilia, resulting in increased NLR. However in NHB individuals, innate immunity may be naturally lower with BEN, resulting in lower NLR (13).

Similarly, the degree of inflammation did not significantly vary by neighborhood CDI even though individuals from the most disadvantaged neighborhoods presented with more late-stage lung cancer compared to those residing in the least disadvantaged neighborhoods. After controlling for stage of cancer, neighborhood CDI, BMI and age categories, and smoking history, NHB race was associated with greater than a two unit decrease in NLR compared to NHW. Social cohesion, or the feeling of connectedness, solidarity and a sense of belonging within a community, may help explain these findings (51). Amongst groups and individuals in disadvantaged neighborhoods, social cohesion may have a positive impact on inflammation and health outcomes (52–54). Studies have shown that members of groups with a history of discrimination may have better health when living in areas of the same racial/ethnic density (52). In our study, the majority of NHB individuals (66%) lived in neighborhoods where Blacks made up ≥75% of the population, and the majority of NHW individuals lived in neighborhoods where racial composition was <75% Black. Racial density may lead to less stigma when amongst one’s own racial group or social class (52) and the protective effects of social support and feelings of belonging that provide benefit from the effects of racism and discrimination (51). In a study investigating neighborhood social cohesion and inflammation (measured by CRP and IL-6), there was an inverse association between social cohesion and CRP or IL-6 (53). While our study did not measure neighborhood social cohesion, this may be one mechanism explaining the lower degree of inflammation in NHB individuals, and the lower prevalence of inflammation in NHB individuals and in those from more disadvantaged neighborhoods, even in the presence of more advanced lung cancer stage at diagnosis. Similarly, resilience, or a person’s ability to adapt to an adverse situation and recover (55), may help to attenuate the negative effects of stressors, including SES and childhood adverse life experiences, on inflammatory markers (55, 56) and may be another mechanism for lower inflammation in NHB individuals with lung cancer from disadvantaged neighborhoods.

Finally, we confirm that a large proportion of NHB individuals live in the most disadvantaged neighborhoods in the larger Chicago metropolitan area and present with higher prevalence of late-stage cancer compared to NHW individuals. Additionally, a larger proportion of those who live in the most disadvantaged neighborhoods present with late-stage cancer at a significantly younger age compared to those who live in the least disadvantaged neighborhoods, which may suggest limited access to care or resources (17, 57, 58). The racially patterned socioeconomic disadvantage that occurs in this region may impact stage of cancer at diagnosis and disparities in lung cancer outcomes.

### Limitations

Although power analysis was completed *a priori* using pilot data for NLR differences by race, the study may not have been adequately powered to detect differences by neighborhood CDI. This was a cross-sectional study and only investigated inflammation at one time point near or on the cancer diagnosis date, thus causation cannot be inferred. Differences in inflammation by race and neighborhood may present longitudinally or as the cancer is treated. We used NLR as a marker of inflammation due to its clinical availability, but NLR may only be weakly correlated with CRP, another inflammatory biomarker (36). NLR is a sensitive marker of acute and/or chronic inflammation associated with infectious and non-infectious diseases and reflects the relationship between innate and adaptive immunity (13), but it remains unknown whether NLR is an appropriate marker of inflammation when examining racial differences, especially due to the presence of BEN, and whether NLR is impacted by social stress as measured by CDI in this population. In addition, we acknowledge the importance of adjusting for white blood cells when looking at the association between NLR and overall and cause-specific mortality, as white blood cells could act as a confounder as they are higher in individuals with higher NLR; however, we did not abstract this information from the electronic medical record. Because an elevated NLR is linked to overall mortality (59) and progression-free and overall survival in lung cancer (7), we cannot exclude a potential selection bias due to racial differences in pre-cancer mortality. As the subjects were obtained from a lung cancer registry from a single urban, tertiary medical center in Cook County, IL, this may represent selection bias as the subjects may not be a representative sample of the overall population with lung cancer. In addition, the inclusion criteria may have biased the sample to those able to access care at the facility and those individuals who receive routine medical care. Finally, we recognize that the association between underweight and elevated NLR in this study may only be a correlation given that we only have one BMI measurement at diagnosis, which does not give us any information about unintended weight loss or malnutrition a person may or may not be experiencing prior to or at diagnosis. Therefore, we cannot establish temporality, and we encourage readers to interpret this finding with caution. Indeed, nutrition risk is associated with increased NLR (60), but nutitional status was not assessed in this study, and BMI at diagnosis is not an indicator of nutritional risk.

## Conclusion

Individuals with lung cancer present with high levels of inflammation upon cancer diagnosis. The degree and prevalence of inflammation, as measured by NLR, at lung cancer diagnosis is significantly lower in NHB compared to NHW individuals and prevalence is significantly lower in those from the most disadvantaged neighborhoods compared to those from the least disadvantaged neighborhoods. While inherently low levels of NLR and protective effects of racially dense neighborhoods may contribute to these results, further research is needed to determine mechanisms that may be contributing to disparities in inflammation amongst NHB and NHW individuals with lung cancer and to establish whether NLR is an appropriate marker to evaluate racial differences in inflammation.

## Data availability statement

The raw data supporting the conclusions of this article will be made available by the authors, without undue reservation.

## Ethics statement

The studies involving human participants were reviewed and approved by University of Illinois Chicago Institutional Review Board and the Rush University Medical Center Institutional Review Board. Written informed consent for participation was not required for this study in accordance with the national legislation and the institutional requirements.

## Author contributions

All authors contributed to the study conception and design. Material preparation, data collection and analysis were performed by CB. The first draft of the manuscript was written by CB and GF and all authors commented on previous versions of the manuscript. All authors read and approved the final manuscript.

## Funding

This study was supported by the University of Illinois Chicago Access to Excellence Fellowship.

## Conflict of interest

The authors declare that the research was conducted in the absence of any commercial or financial relationships that could be construed as a potential conflict of interest.

## Publisher’s note

All claims expressed in this article are solely those of the authors and do not necessarily represent those of their affiliated organizations, or those of the publisher, the editors and the reviewers. Any product that may be evaluated in this article, or claim that may be made by its manufacturer, is not guaranteed or endorsed by the publisher.

## References

[1] Group UCSW. U.S. Cancer statistics data visualizations tool, based on 2021 submission data (1999-2019). (Atlanta, GA: U.S. Department of Health and Human Services, Centers for Disease Control and Prevention and National Cancer Institute) (2021). Available at: www.cdc.gov/cancer/dataviz.

[2] Stepanikova I Bateman LB Oates GR. Systemic inflammation in midlife: Race, socioeconomic status, and perceived discrimination. Am J Prev Med (2017) 52(1S1):S63–76. doi: 10.1016/j.amepre.2016.09.026 PMC5319849 27989295

[3] Saini G Ogden A McCullough LE Torres M Rida P Aneja R. Disadvantaged neighborhoods and racial disparity in breast cancer outcomes: the biological link. Cancer Causes Control (2019) 30(7):677–86. doi: 10.1007/s10552-019-01180-4 PMC7043809 31111277

[4] Lee J Taneja V Vassallo R. Cigarette smoking and inflammation: cellular and molecular mechanisms. J Dent Res (2012) 91(2):142–9. doi: 10.1177/0022034511421200 PMC3261116 21876032

[5] Furman D Campisi J Verdin E Carrera-Bastos P Targ S Franceschi C. Chronic inflammation in the etiology of disease across the life span. Nat Med (2019) 25(12):1822–32. doi: 10.1038/s41591-019-0675-0 PMC7147972 31806905

[6] O'Byrne KJ Dalgleish AG. Chronic immune activation and inflammation as the cause of malignancy. Br J Cancer. (2001) 85(4):473–83. doi: 10.1054/bjoc.2001.1943 PMC2364095 11506482

[7] Go SI Park MJ Song HN Kang MH Park HJ Jeon KN. Sarcopenia and inflammation are independent predictors of survival in male patients newly diagnosed with small cell lung cancer. Support Care Cancer (2016) 24(5):2075–84. doi: 10.1007/s00520-015-2997-x 26546456

[8] Suzuki R Lin SH Wei X Allen PK Welsh JW Byers LA. Prognostic significance of pretreatment total lymphocyte count and neutrophil-to-lymphocyte ratio in extensive-stage small-cell lung cancer. Radiother Oncol (2018) 126(3):499–505. doi: 10.1016/j.radonc.2017.12.030 29398150 PMC5856620

[9] Templeton AJ McNamara MG Šeruga B Vera-Badillo FE Aneja P Ocaña A. Prognostic role of neutrophil-to-lymphocyte ratio in solid tumors: a systematic review and meta-analysis. J Natl Cancer Inst (2014) 106(6):dju124. doi: 10.1093/jnci/dju124 24875653

[10] Marrugal Á Ojeda L Paz-Ares L Molina-Pinelo S Ferrer I. Proteomic-based approaches for the study of cytokines in lung cancer. Dis Markers (2016) 2016:2138627. doi: 10.1155/2016/2138627 27445423 PMC4944034

[11] Walsh SR Cook EJ Goulder F Justin TA Keeling NJ. Neutrophil-lymphocyte ratio as a prognostic factor in colorectal cancer. J Surg Oncol (2005) 91(3):181–4. doi: 10.1002/jso.20329 16118772

[12] Biswas T Gawdi R Jindal C Iyer S Kang KH Bajor D. Pretreatment neutrophil-to-lymphocyte ratio as an important prognostic marker in stage III locally advanced non-small cell lung cancer: confirmatory results from the PROCLAIM phase III clinical trial. J Thorac Dis (2021) 13(10):5617–26. doi: 10.21037/jtd-21-1018 PMC8575807 34795912

[13] Zahorec R. Neutrophil-to-lymphocyte ratio, past, present and future perspectives. Bratisl Lek Listy (2021) 122(7):474–88. doi: 10.4149/BLL_2021_078 34161115

[14] Guthrie GJ Charles KA Roxburgh CS Horgan PG McMillan DC Clarke SJ. The systemic inflammation-based neutrophil-lymphocyte ratio: experience in patients with cancer. Crit Rev Oncol Hematol (2013) 88(1):218–30. doi: 10.1016/j.critrevonc.2013.03.010 23602134

[15] Krieger N. Refiguring "race": epidemiology, racialized biology, and biological expressions of race relations. Int J Health Serv (2000) 30(1):211–6. doi: 10.2190/672J-1PPF-K6QT-9N7U 10707306

[16] Solar O Irwin A World Health Organization. A conceptual framework for action on the social determinants of health. social determinants of health discussion paper 2 (Policy and practice) (Geneva: World Health Organization). (2010).

[17] Link BG Phelan J. Social conditions as fundamental causes of disease. J Health Soc Behav (1995) Forty Years of Medical Sociology: The State of the Art and Directions for the Future, 80–94. doi: 10.2307/2626958 7560851

[18] Cockerham WC Hamby BW Oates GR. The social determinants of chronic disease. Am J Prev Med (2017) 52(1S1):S5–S12. doi: 10.1016/j.amepre.2016.09.010 27989293 PMC5328595

[19] Danos DM Ferguson TF Simonsen NR Leonardi C Yu Q Wu XC. Neighborhood disadvantage and racial disparities in colorectal cancer incidence: a population-based study in Louisiana. Ann Epidemiol (2018) 28(5):316–21.e2. doi: 10.1016/j.annepidem.2018.02.004 29678311 PMC6138869

[20] Sampson RJ Raudenbush SW Earls F. Neighborhoods and violent crime: a multilevel study of collective efficacy. Science (1997) 277(5328):918–24. doi: 10.1126/science.277.5328.918 9252316

[21] Sampson R Wilson W. Toward a theory of race, crime, and urban inequality. In: Hagan J Ruth P, editors. Crime and inequality. Stanford, CA: Stanford University Press (1995). p. 37–56.

[22] Sampson RJ Morenoff JD TG-R. Assessing”neighborhood effects”: social processes and new directions in research. Annu Rev Sociol (2002) 28:443–78. doi: 10.1146/annurev.soc.28.110601.141114

[23] Schulz AJ Mentz G Lachance L Zenk SN Johnson J Stokes C. Do observed or perceived characteristics of the neighborhood environment mediate associations between neighborhood poverty and cumulative biological risk? Health Place (2013) 24:147–56. doi: 10.1016/j.healthplace.2013.09.005 PMC3837295 24100238

[24] Schulz AJ Zenk SN Israel BA Mentz G Stokes C Galea S. Do neighborhood economic characteristics, racial composition, and residential stability predict perceptions of stress associated with the physical and social environment? findings from a multilevel analysis in Detroit. J Urban Health (2008) 85(5):642–61. doi: 10.1007/s11524-008-9288-5 PMC2527427 18481182

[25] Gomez SL Shariff-Marco S DeRouen M Keegan TH Yen IH Mujahid M. The impact of neighborhood social and built environment factors across the cancer continuum: Current research, methodological considerations, and future directions. Cancer (2015) 121(14):2314–30. doi: 10.1002/cncr.29345 PMC4490083 25847484

[26] Broyles ST Staiano AE Drazba KT Gupta AK Sothern M Katzmarzyk PT. Elevated c-reactive protein in children from risky neighborhoods: evidence for a stress pathway linking neighborhoods and inflammation in children. PLoS One (2012) 7(9):e45419. doi: 10.1371/journal.pone.0045419 23049799 PMC3458094

[27] Keita AD Judd SE Howard VJ Carson AP Ard JD Fernandez JR. Associations of neighborhood area level deprivation with the metabolic syndrome and inflammation among middle- and older- age adults. BMC Public Health (2014) 14:1319. doi: 10.1186/1471-2458-14-1319 25539758 PMC4364504

[28] Ribeiro AI Fraga S Kelly-Irving M Delpierre C Stringhini S Kivimaki M. Neighbourhood socioeconomic deprivation and allostatic load: a multi-cohort study. Sci Rep (2019) 9(1):8790. doi: 10.1038/s41598-019-45432-4 31217447 PMC6584573

[29] Kelley-Hedgepeth A Lloyd-Jones DM Colvin A Matthews KA Johnston J Sowers MR. Ethnic differences in c-reactive protein concentrations. Clin Chem (2008) 54(6):1027–37. doi: 10.1373/clinchem.2007.098996 18403563

[30] Khera A McGuire DK Murphy SA Stanek HG Das SR Vongpatanasin W. Race and gender differences in c-reactive protein levels. J Am Coll Cardiol (2005) 46(3):464–9. doi: 10.1016/j.jacc.2005.04.051 16053959

[31] Richman AD. Concurrent social disadvantages and chronic inflammation: The intersection of race and ethnicity, gender, and socioeconomic status. J Racial Ethn Health Disparities. (2018) 5(4):787–97. doi: 10.1007/s40615-017-0424-3 28849408

[32] Zahodne LB Kraal AZ Zaheed A Farris P Sol K. Longitudinal effects of race, ethnicity, and psychosocial disadvantage on systemic inflammation. SSM Popul Health (2019) 7:100391. doi: 10.1016/j.ssmph.2019.100391 31193191 PMC6520605

[33] Permuth JB Clark Daly A Jeong D Choi JW Cameron ME Chen DT. Racial and ethnic disparities in a state-wide registry of patients with pancreatic cancer and an exploratory investigation of cancer cachexia as a contributor to observed inequities. Cancer Med (2019) 8(6):3314–24. doi: 10.1002/cam4.2180 PMC6558500 31074202

[34] Azab B Camacho-Rivera M Taioli E. Average values and racial differences of neutrophil lymphocyte ratio among a nationally representative sample of united states subjects. PLoS One (2014) 9(11):e112361. doi: 10.1371/journal.pone.0112361 25375150 PMC4223021

[35] Howard R Kanetsky PA Egan KM. Exploring the prognostic value of the neutrophil-to-lymphocyte ratio in cancer. Sci Rep (2019) 9(1):19673. doi: 10.1038/s41598-019-56218-z 31873162 PMC6928022

[36] Howard R Scheiner A Kanetsky PA Egan KM. Sociodemographic and lifestyle factors associated with the neutrophil-to-lymphocyte ratio. Ann Epidemiol (2019) 38:11–21.e6. doi: 10.1016/j.annepidem.2019.07.015 31481293 PMC8653546

[37] Vidal AC Howard LE de Hoedt A Cooperberg MR Kane CJ Aronson WJ. Neutrophil, lymphocyte and platelet counts, and risk of prostate cancer outcomes in white and black men: results from the SEARCH database. Cancer Causes Control (2018) 29(6):581–8. doi: 10.1007/s10552-018-1031-2 PMC5940530 29663110

[38] Coulon SJ Velasco-Gonzalez C Scribner R Park CL Gomez R Vargas A. Racial differences in neighborhood disadvantage, inflammation and metabolic control in black and white pediatric type 1 diabetes patients. Pediatr Diabetes (2017) 18(2):120–7. doi: 10.1111/pedi.12361 PMC4949146 26783014

[39] Cozier YC Albert MA Castro-Webb N Coogan PF Ridker P Kaufman HW. Neighborhood socioeconomic status in relation to serum biomarkers in the black women's health study. J Urban Health (2016) 93(2):279–91. doi: 10.1007/s11524-016-0034-0 PMC4835359 27000125

[40] Petersen KL Marsland AL Flory J Votruba-Drzal E Muldoon MF Manuck SB. Community socioeconomic status is associated with circulating interleukin-6 and c-reactive protein. Psychosom Med (2008) 70(6):646–52. doi: 10.1097/PSY.0b013e31817b8ee4 18606725

[41] Fedewa MV Das BM Forehand RL Evans EM. Area-level socioeconomic status, adiposity, physical activity, and inflammation in young adults, 2013. Prev Chronic Dis (2014) 11:E130. doi: 10.5888/pcd11.140090 25078567 PMC4124040

[42] Xiao J Caan BJ Cespedes Feliciano EM Meyerhardt JA Kroenke CH Baracos VE. The association of medical and demographic characteristics with sarcopenia and low muscle radiodensity in patients with nonmetastatic colorectal cancer. Am J Clin Nutr (2019) 109(3):615–25. doi: 10.1093/ajcn/nqy328 PMC6408202 30850836

[43] Feliciano EMC Kroenke CH Meyerhardt JA Prado CM Bradshaw PT Kwan ML. Association of systemic inflammation and sarcopenia with survival in nonmetastatic colorectal cancer: Results from the c SCANS study. JAMA Oncol (2017) 3(12):e172319. doi: 10.1001/jamaoncol.2017.2319 28796857 PMC5824285

[44] Peterson CE Rauscher GH Johnson TP Kirschner CV Freels S Barrett RE. The effect of neighborhood disadvantage on the racial disparity in ovarian cancer-specific survival in a large hospital-based study in cook county, illinois. Front Public Health (2015) 3:8. doi: 10.3389/fpubh.2015.00008 25657992 PMC4302660

[45] Templeton AJ Ace O McNamara MG Al-Mubarak M Vera-Badillo FE Hermanns T. Prognostic role of platelet to lymphocyte ratio in solid tumors: a systematic review and meta-analysis. Cancer Epidemiol Biomarkers Prev (2014) 23(7):1204–12. doi: 10.1158/1055-9965.EPI-14-0146 24793958

[46] Gupta D Lis CG. Pretreatment serum albumin as a predictor of cancer survival: a systematic review of the epidemiological literature. Nutr J (2010) 9:69. doi: 10.1186/1475-2891-9-69 21176210 PMC3019132

[47] Zhang Y Brodin NP Ohri N Thibaud S Kaubisch A Kinkhabwala M. Association between neutrophil-lymphocyte ratio, socioeconomic status, and ethnic minority with treatment outcome in hepatocellular carcinoma. Hepatol Int (2019) 13(5):609–17. doi: 10.1007/s12072-019-09965-0 31372942

[48] Kim SI Kim HS Kim TH Suh DH Kim K No JH. Impact of underweight after treatment on prognosis of advanced-stage ovarian cancer. J Immunol Res (2014) 2014:349546. doi: 10.1155/2014/349546 25050383 PMC4094724

[49] Scilla KA Bentzen SM Lam VK Mohindra P Nichols EM Vyfhuis MA. Neutrophil-lymphocyte ratio is a prognostic marker in patients with locally advanced (Stage IIIA and IIIB) non-small cell lung cancer treated with combined modality therapy. Oncologist (2017) 22(6):737–42. doi: 10.1634/theoncologist.2016-0443 PMC5469587 28533476

[50] Atallah-Yunes SA Ready A Newburger PE. Benign ethnic neutropenia. Blood Rev (2019) 37:100586. doi: 10.1016/j.blre.2019.06.003 31255364 PMC6702066

[51] Bécares L Shaw R Nazroo J Stafford M Albor C Atkin K. Ethnic density effects on physical morbidity, mortality, and health behaviors: a systematic review of the literature. Am J Public Health (2012) 102(12):e33–66. doi: 10.2105/AJPH.2012.300832 PMC3519331 23078507

[52] Pickett KE Wilkinson RG. People like us: ethnic group density effects on health. Ethn Health (2008) 13(4):321–34. doi: 10.1080/13557850701882928 18701992

[53] Neergheen VL Topel M Van Dyke ME Sullivan S Pemu PE Gibbons GH. Neighborhood social cohesion is associated with lower levels of interleukin-6 in African American women. Brain Behav Immun (2019) 76:28–36. doi: 10.1016/j.bbi.2018.10.008 30686334 PMC6370481

[54] Kawachi I BL. Social cohesion, social capital, and health. In: Berkman LFKI, editor. Social epidemiology. New York, NY: Oxford University Press (2000).

[55] Dantzer R Cohen S Russo SJ Dinan TG. Resilience and immunity. Brain Behav Immun (2018) 74:28–42. doi: 10.1016/j.bbi.2018.08.010 30102966 PMC6545920

[56] Gouin JP Caldwell W Woods R Malarkey WB. Resilience resources moderate the association of adverse childhood experiences with adulthood inflammation. Ann Behav Med (2017) 51(5):782–6. doi: 10.1007/s12160-017-9891-3 28281135

[57] Berger M Lund MJ Brawley OW. Racial disparities in lung cancer. Curr Probl Cancer. (2007) 31(3):202–10. doi: 10.1016/j.currproblcancer.2007.02.002 17543948

[58] Kim S Chukwudozie B Calhoun E. Sociodemographic characteristics, distance to the clinic, and breast cancer screening results. J Health Dispar Res Pract (2013) 6(1):70. 24466505 PMC3898539

[59] Song M Graubard BI Rabkin CS Engels EA. Neutrophil-to-lymphocyte ratio and mortality in the united states general population. Sci Rep (2021) 11(1):464. doi: 10.1038/s41598-020-79431-7 33431958 PMC7801737

[60] Siqueira JM Soares JDP Borges TC Gomes TLN Pimentel GD. High neutrophil to lymphocytes ratio is associated with nutritional risk in hospitalised, unselected cancer patients: a cross-sectional study. Sci Rep (2021) 11(1):17120. doi: 10.1038/s41598-021-96586-z 34429466 PMC8384836

