# Supplementary figures and images for: Disparities in inflammation between non-Hispanic black and white individuals with lung cancer in the Greater Chicago Metropolitan area
Source: Front Immunol. 2022 Dec 5;13:1008674. doi: 10.3389/fimmu.2022.1008674 (PMC9760905; doi:10.3389/fimmu.2022.1008674)

**Supplementary Figure 1: Flow Chart Depicting the Inclusion and Exclusion of Subjects**

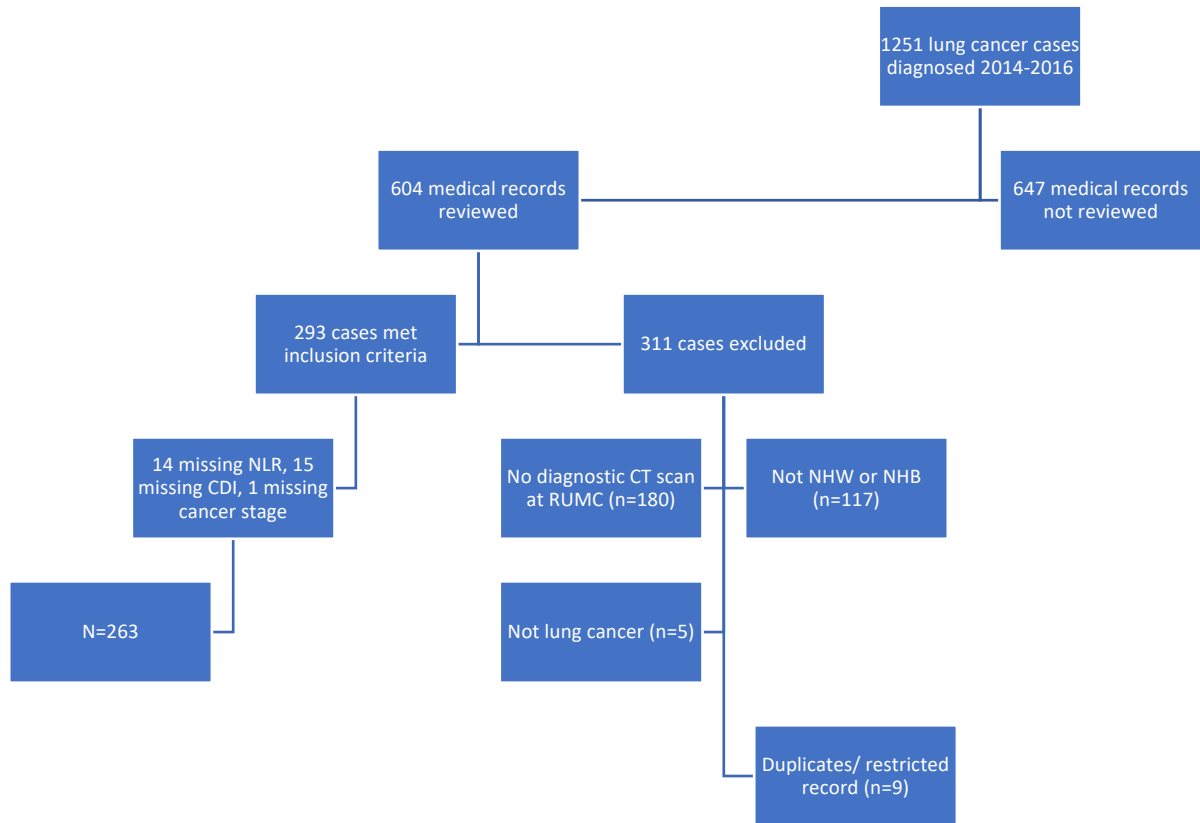

Supplement: Supplementary file 1 [file Image_1.pdf]
