# Supplementary material for: Disparities in inflammation between non-Hispanic black and white individuals with lung cancer in the Greater Chicago Metropolitan area
Source: Front Immunol. 2022 Dec 5;13:1008674. doi: 10.3389/fimmu.2022.1008674 (PMC9760905; doi:10.3389/fimmu.2022.1008674)

**Supplementary Figure 2: Distribution of NLR in the Overall Sample of Individuals with Lung Cancer**

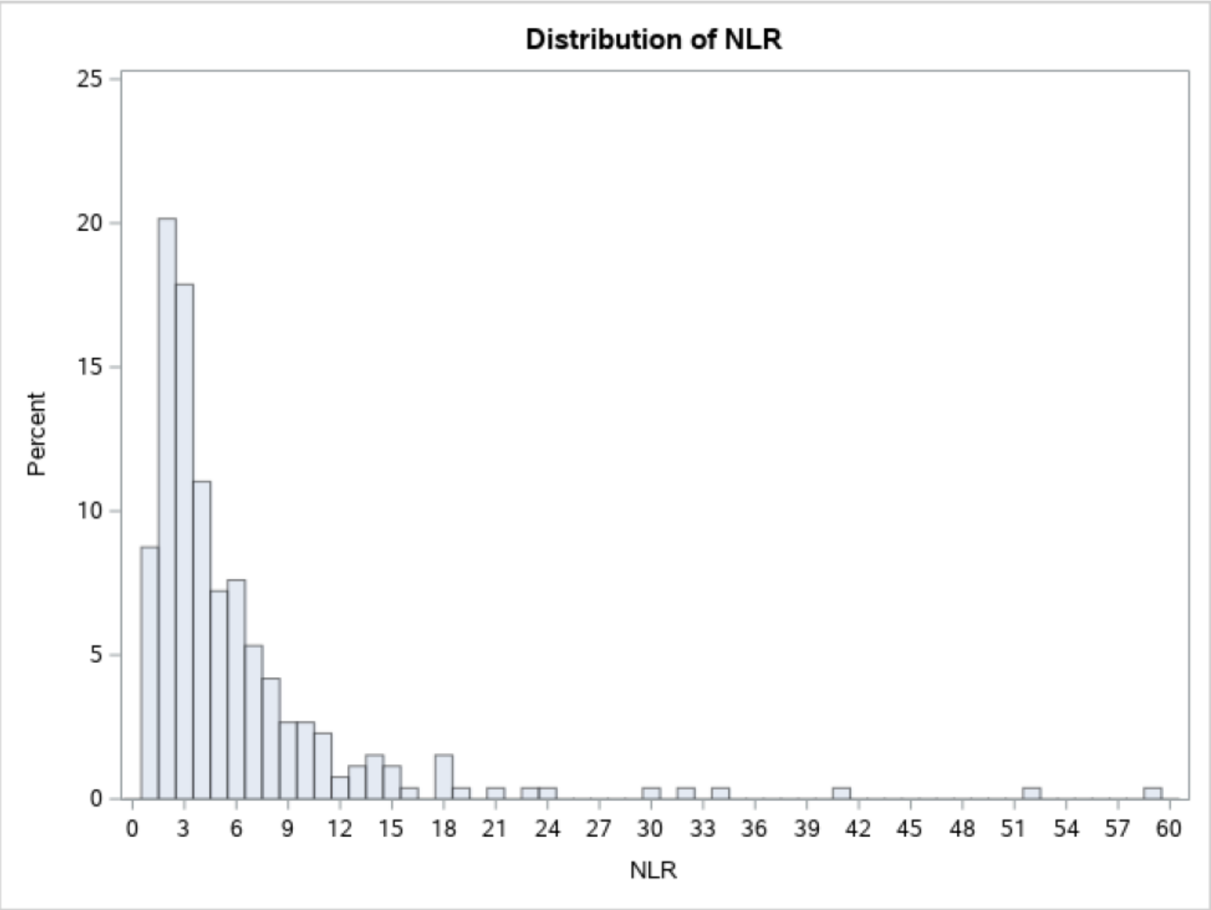

Supplement: Supplementary file 2 [file Image_2.pdf]
