# Supplementary material for: Disparities in inflammation between non-Hispanic black and white individuals with lung cancer in the Greater Chicago Metropolitan area
Source: Front Immunol. 2022 Dec 5;13:1008674. doi: 10.3389/fimmu.2022.1008674 (PMC9760905; doi:10.3389/fimmu.2022.1008674)
Supplement: Supplementary file 3 [file Image_3.pdf]

Supplementary Figure 3: Distribution of NLR in Non-Hispanic White and Non-Hispanic Black Individuals with Lung Cancer[1]

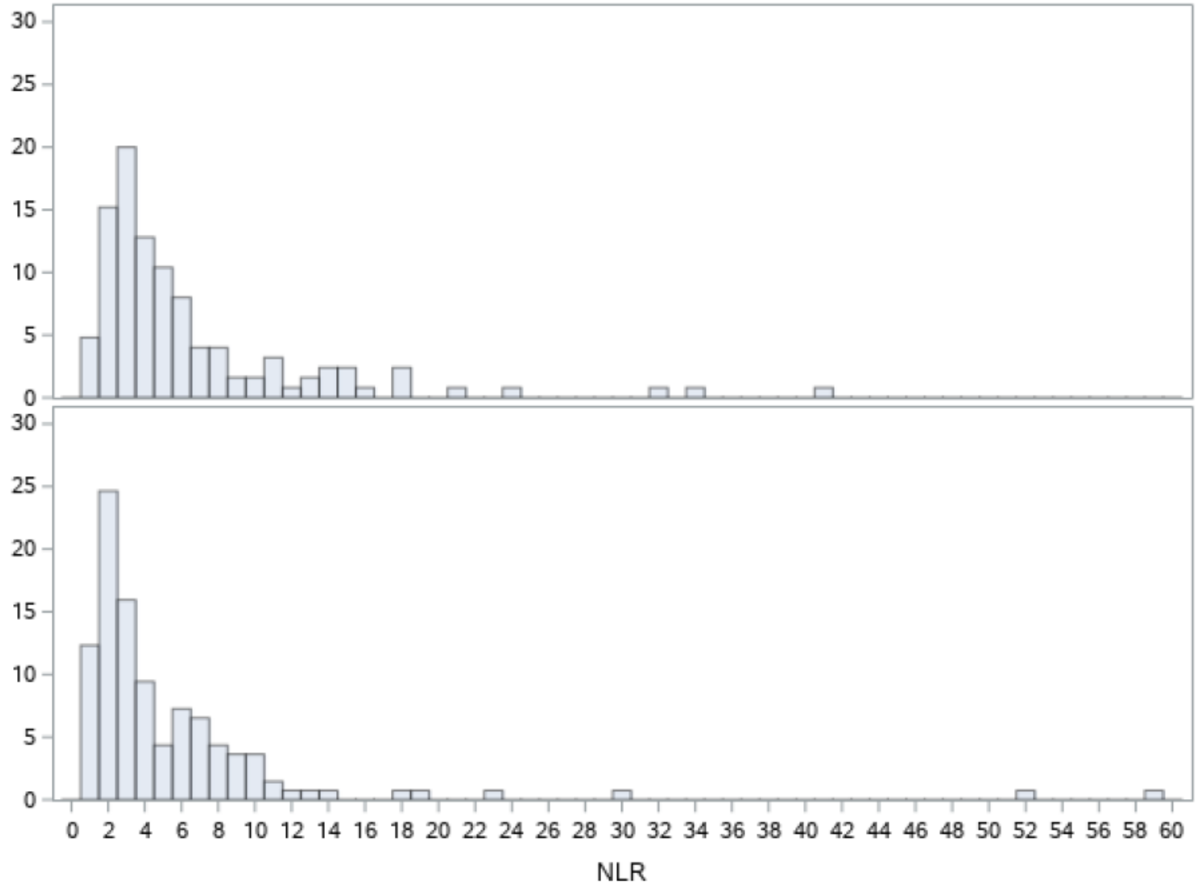

[1] Top figure represents NLR distribution in Non-Hispanic White individuals while bottom figure represents NLR distribution in Non-Hispanic Black individuals
