# Supplementary material for: Disparities in inflammation between non-Hispanic black and white individuals with lung cancer in the Greater Chicago Metropolitan area
Source: Front Immunol. 2022 Dec 5;13:1008674. doi: 10.3389/fimmu.2022.1008674 (PMC9760905; doi:10.3389/fimmu.2022.1008674)
Supplement: Supplementary file 4 [file Table_1.docx]

**Supplementary Table 1: Additional Demographic Variables of Subjects with Lung Cancer Overall and by Race (n=263)^1^**

| **Overall Sample** |  |  | **NHB (n=138)** | **NHW (n=125)** | p-value |
| --- | --- | --- | --- | --- | --- |
| BMI Group |  |  |  |  |  |
| Underweight (<18.5 kg/m^2^) | 22 (8.37) | **<0.0001** | 15 (5.70) | 7 (2.66) | 0.35 |
| Normal  (18.5-<25 kg/m^2^) | 89 (33.84) |  | 47 (17.87) | 42 (15.97) |  |
| Overweight  (25-<30 kg/m^2^) | 95 (36.12) |  | 50 (36.23) | 45 (36.00) |  |
| Obese  (>30 kg/m^2^) | 57 (21.67) |  | 26 (18.84) | 31 (24.80) |  |
| Age Group |  |  |  |  |  |
| <65 years | 87 (33.08) | **<0.0001** | 52 (37.68) | 35 (28.00) | 0.10 |
| ≥65 years | 176 (66.92) |  | 86 (62.32) | 90 (72.00) |  |
| Alcohol History |  |  |  |  |  |
| Current/Former | 170 (65.89) | **<0.0001** | 84 (62.22) | 86 (69.92) | 0.19 |
| Never | 88 (34.11) |  | 51 (37.78) | 37 (30.08) |  |
| Neighborhoods with <75% Black individuals | 171 (65.02) | **<0.0001** | 47 (34.06) | 124 (99.20) | **<0.0001** |
| Neighborhoods with ≥75% Black individuals | 92 (34.98) |  | 91 (65.94) | 1 (0.80) |  |

^1^Values are means + SDs or n (%).

NHB= Non-Hispanic Black; NHW= Non-Hispanic White; BMI= Body Mass Index; kg= kilograms; m^2^= meters squared
