# Supplementary material for: Disparities in inflammation between non-Hispanic black and white individuals with lung cancer in the Greater Chicago Metropolitan area
Source: Front Immunol. 2022 Dec 5;13:1008674. doi: 10.3389/fimmu.2022.1008674 (PMC9760905; doi:10.3389/fimmu.2022.1008674)
Supplement: Supplementary file 5 [file Table_2.docx]

**Supplementary Table 2: Demographics and Inflammatory Biomarkers by Neighborhood Concentrated Disadvantage in Subjects with Lung Cancer (n=263)^1^**

|  | **Least Disadvantaged Neighborhoods (n=128)** | | **Most Disadvantaged Neighborhoods (n=135)** | | **p-value** |
| --- | --- | --- | --- | --- | --- |
| Age (years) | 70.39 + 8.94 | | 68.01 + 10.49 | | **0.05** |
| BMI (kg/m^2^) | 25.75 + 5.11 | | 26.75 + 6.26 | | 0.16 |
| NLR | 5.95 + 5.47 | | 6.03 + 8.26 | | 0.06* |
| NLR by CDI Quartile | Q1 | Q2 | Q3 | Q4 | 0.26 |
|  | 6.18 + 5.93 | 5.72 + 5.00 | 5.91 + 8.33 | 6.14 + 8.25 |  |
| Race |  | |  | |  |
| NHB | 34 (26.56) | | 104 (77.04) | | **<0.0001** |
| NHW | 94 (73.44) | | 31 (22.96) | |  |
| Sex |  | |  | |  |
| Male | 60 (46.87) | | 77 (57.04) | | 0.10 |
| Female | 68 (53.13) | | 58 (42.96) | |  |
| Cancer Stage |  | |  | |  |
| Early-Stage (0,1,2) | 68 (53.13) | | 40 (29.63) | | **0.0001** |
| Late-Stage (3, 4) | 60 (46.87) | | 95 (70.37) | |  |
| Smoking History |  | |  | |  |
| Current/Former | 117 (91.41) | | 123 (92.48) | | 0.75 |
| Never | 11 (8.59) | | 10 (7.52) | |  |
| BMI Group |  | |  | |  |
| Underweight (<18.5 kg/m^2^) | 11 (8.59) | | 11 (8.15) | | 0.72 |
| Normal  (18.5-<25 kg/m^2^) | 46 (35.94) | | 43 (31.85) | |  |
| Overweight  (25-<30 kg/m^2^) | 47 (36.72) | | 48 (35.56) | |  |
| Obese  (>30 kg/m^2^) | 24 (18.75) | | 33 (24.44) | |  |
| Age Group |  | |  | |  |
| <65 years | 31 (24.22) | | 56 (41.48) | | **0.003** |
| ≥65 years | 97 (75.78) | | 79 (58.52) | |  |
| Neighborhoods with <75% Black individuals | 122 (95.31) | | 49 (36.30) | | **<0.0001** |
| Neighborhoods with ≥75% Black individuals | 6 (4.69) | | 86 (63.70) | |  |

^1^Values are means + SDs or n (%).

*Mann Whitney U test as NLR is not normally distributed in this sample. T-test use for other continuous variables and Pearson’s chi square test used for categorical variables.

BMI= body mass index; kg= kilograms; m^2^= meters squared; NLR= neutrophil to lymphocyte ratio; NHB=Non-Hispanic Black; NHW= Non-Hispanic White; Q1= quartile 1 for neighborhood concentrated disadvantage index; Q2= quartile 2; Q3= quartile 3; Q4= quartile 4
