# Supplementary material for: Disparities in inflammation between non-Hispanic black and white individuals with lung cancer in the Greater Chicago Metropolitan area
Source: Front Immunol. 2022 Dec 5;13:1008674. doi: 10.3389/fimmu.2022.1008674 (PMC9760905; doi:10.3389/fimmu.2022.1008674)
Supplement: Supplementary file 6 [file Table_3.docx]

**Supplementary Table 3: NLR by Concentrated Disadvantage in Non-Hispanic Black individuals only and in Non-Hispanic Whites only***

|  | **Least Disadvantage** | **Most Disadvantage** | **p-value** |
| --- | --- | --- | --- |
| NHB only (n=138) | 4.72 + 3.56 | 5.75 + 8.33 | 0.71 |
| NHW only (n=125) | 6.40 + 5.97 | 6.95 + 8.09 | 0.59 |

*^1^Values are means + SDs; Mann Whitney U test as NLR is not normally distributed in this sample

NHB= Non-Hispanic Blacks; NHW= Non-Hispanic Whites
