# Supplementary material for: Disparities in inflammation between non-Hispanic black and white individuals with lung cancer in the Greater Chicago Metropolitan area
Source: Front Immunol. 2022 Dec 5;13:1008674. doi: 10.3389/fimmu.2022.1008674 (PMC9760905; doi:10.3389/fimmu.2022.1008674)
Supplement: Supplementary file 7 [file Table_4.docx]

**Supplementary Table 4: Additional NLR Analyses by Stage of Cancer, Histology Sub-Type, Smoking Status, BMI and Age Categories***

|  | **Early-stage (n=108)** | | | | | **Late-stage (n=155)** | | | | | **p-value** |
| --- | --- | --- | --- | --- | --- | --- | --- | --- | --- | --- | --- |
| **NLR by Stage of Cancer** | 5.01 + 5.17 | | | | | 6.67 + 8.02 | | | | | **0.04** |
|  | NHB (n=40) | NHW (n=68) | | | p-value | NHB (n=98) | | NHW (n=57) | p-value | |  |
|  | 3.58 + 2.59 | 5.86 + 6.07 | | | **0.02** | 6.29 + 8.57 | | 7.34 + 7.00 | | **0.03** |  |
|  |  | | | | |  | | | | |  |
|  | **NSCLC (n=224)** | | | | | **SCLC (n=7)** | | | | |  |
| **NLR by Histology** | 5.72 + 6.53 | | | | | 8.85 + 10.58 | | | | | 0.24 |
|  |  | | | | |  | | | | |  |
|  | **Current/Former (n=240)** | | | | | **Never (n=21)** | | | | |  |
| **NLR by Smoking Status** | 5.82 + 6.39 | | | | | 5.66 + 4.46 | | | | | 0.68 |
|  | NHB (n=126) | | NHW (n=114) | |  |  | | | | |  |
|  | 5.06 + 5.99 | | 6.66 + 6.73 | | **0.006** |  | | | | |  |
|  |  | |  | |  |  | | | | |  |
|  | Least Disadvantage  (n=117) | | Most Disadvantage  (n=123) | |  |  | | | | |  |
|  | 6.03 + 5.63 | | 5.61 + 7.03 | | **0.04** |  | | | | |  |
|  |  | | | | |  | | | | |  |
|  | **Underweight** (n=22) | | | **Normal Weight** (n=89) | | **Overweight** (n=95) | **Obese** (n=57) | | | |  |
| **NLR by BMI Category** | 10.66 + 12.03 | | | 6.22 + 6.85 | | 5.13 + 4.89 | 5.28 + 7.26 | | | | **0.006** |
|  |  | | | | |  | | | | |  |
|  | **< 65 years** | | | | | **>65 years** | | | | |  |
| **NLR by Age Category** | 6.60 + 9.17 | | | | | 5.69 + 5.69 | | | | | 0.60 |

*Values are means + SDs; Mann Whitney U test as NLR is not normally distributed in this sample

NLR= neutrophil to lymphocyte ratio; NHB=Non-Hispanic Black; NHW= Non-Hispanic White; NSCLC= non-small cell lung cancer; SCLC= small cell lung cancer; BMI= body mass index; kg= kilograms; m^2^= meters squared; CDI= concentrated disadvantage index; Q1= quartile 1; Q2= quartile 2; Q3= quartile 3; Q4= quartile 4
